# Economic burden of typhoid fever by antimicrobial resistance in India: a modelling study 2023

**DOI:** 10.1016/j.lansea.2026.100748

**Published:** 2026-03-20

**Authors:** Vijayalaxmi V. Mogasale, Jacob John, Arindam Ray, Habib Hasan Farooqui, Vittal Mogasale, Raymond Hutubessy, Bhim Gopal Dhoubhadel, W John Edmunds, Andrew Clark, Kaja Abbas

**Affiliations:** a Department of Infectious Disease Epidemiology and Dynamics, London School of Hygiene & Tropical Medicine, London, UK; b School of Tropical Medicine and Global Health, Nagasaki University, Nagasaki, Japan; c Institute of Tropical Medicine, Nagasaki University, Nagasaki, Japan; d Department of Community Health, Christian Medical College, Vellore, India; e Department of Infectious Disease & Vaccine Delivery, Gates Foundation, New Delhi, India; f College of Medicine, Qatar University, Doha, Qatar; g Graduate School of Public Health, Yonsei University, Seoul, Republic of Korea; h Performance, Finance and Delivery Department, World Health Organisation, Geneva, Switzerland; i Department of Health Services Research and Policy, London School of Hygiene & Tropical Medicine, London, UK; j Public Health Foundation of India, New Delhi, India; k National Institute of Infectious Diseases, Japan Institute for Health Security, Tokyo, Japan

**Keywords:** Typhoid fever, Antimicrobial resistance, Economic burden, Societal perspective, Government perspective, Cost-of-illness, Catastrophic expenditures, Premature deaths, Vaccine introduction

## Abstract

**Background:**

Typhoid fever and rising antimicrobial resistance contribute towards substantial morbidity in India. Introduction of typhoid conjugate vaccine in national immunisation schedule is under consideration to address this growing disease burden. In this study we estimated the economic burden of typhoid fever for 2023, disaggregated by age, provincial states of India, and fluoroquinolone resistance, from societal and government perspectives, to support national vaccination policy.

**Methods:**

We developed a decision-tree model using Indian empirical data on typhoid epidemiology, care-seeking, clinical outcomes, and estimated direct and indirect costs for hospitalised and non-hospitalised typhoid fever patients. To reflect age-specific uncertainty in hospitalisation patterns and resulting economic burden, we used two primary scenarios. We estimated productivity losses due to premature mortality using the human capital approach, with the friction-cost approach as an alternative. We assessed uncertainty through probabilistic sensitivity analysis.

**Findings:**

The economic burden of typhoid fever in India in 2023 was estimated at INR 123.0 billion (95% UI 76.7–215.5; US$ 1.5 billion, 0.9–2.6), including a cost of INR 13.0 billion (6.6–27.0; US$ 157 million, 80–326) to the public health system (government perspective). Fluoroquinolone-resistant infections accounted for 87% of total costs. Children under ten years of age incurred the highest economic burden, contributing over half of the total costs. Households bore 91% of expenses, and 70,000 families faced catastrophic health expenditure. Maharashtra, Uttar Pradesh, Andhra Pradesh (including Telangana), Tamil Nadu, and West Bengal were the states estimated to account for 51% of the national costs. Productivity loss was INR 42.6 billion (15.7–111.1; US$ 515 million) based on the human capital approach and declined by 99.8% under the friction-cost approach.

**Interpretation:**

Typhoid fever imposes a significant economic burden in India, shaped by fluoroquinolone resistance, children less than ten years of age, and high-burden provincial states of the country, resulting in considerable household financial strain. Our findings provide key evidence to support the introduction of the typhoid conjugate vaccine, enhance antimicrobial resistance control, and guide national health financing policies.

**Funding:**

WISE programme; International Vaccine Institute; 10.13039/501100023693Vaccine Impact Modelling Consortium; Japan Agency for Medical Research and Development.

**Research in context**

**Evidence before this study**

We conducted a PubMed search on November 23, 2025, using the terms (typhoid OR “enteric fever”) AND (cost OR expenditure OR economic) AND India, with no language or date restrictions. This search yielded 194 results, which we screened by examining their titles, abstracts, and reference lists. We found limited information regarding the economic burden of typhoid fever in India. The available empirical data include a cost-of-illness study conducted in a Delhi urban slum from 1995 to 1997, and two studies conducted in a Kolkata urban slum between 2003 and 2006. Additionally, a recent multisite study from the Surveillance for Enteric Fever in India (SEFI) network, covering 2017 to 2019, examined household costs associated with hospitalisation and instances of catastrophic health spending. We also found a systematic review of cost-of-illness studies on typhoid fever. Previous studies in India have largely focused on site-specific cost-of-illness estimates or on the cost-effectiveness of typhoid vaccination, with limited evidence quantifying the national economic burden of typhoid fever by provincial state of the country and age. Overall, the available evidence is fragmented, geographically limited, and does not provide nationally representative estimates of the economic burden needed to inform national decisions on introducing the typhoid conjugate vaccine (TCV) or on strategies to address antimicrobial resistance (AMR) in India.

**Added value of this study**

Our study presents a comprehensive and nationally representative economic burden estimate for typhoid fever in India, disaggregated by provincial state, age group, and AMR status. Our decision-tree model incorporated the most recent epidemiological data, updated patterns of AMR, and detailed cost-of-illness evidence from both hospitalised and non-hospitalised patients to quantify the economic burden from both societal and governmental perspectives. Our analysis indicates that fluoroquinolone-resistant infections drive the economic burden of typhoid fever in India, disproportionately affecting young children and placing a significant financial burden on families. We present our estimates disaggregated by state, age group, treatment-seeking behaviour, and AMR profile, addressing a crucial evidence gap and directly informing national and regional decisions on the introduction of TCV, AMR control measures, and policies to protect against financial risk.

**Implications of all the available evidence**

We have highlighted that typhoid fever, combined with AMR, creates a significant economic burden in India. Our findings underscore the need to introduce the TCV, strengthen measures to control AMR, and improve financial protection for vulnerable households. The state- and age-specific estimates we provided offer actionable insights to guide a phased rollout of the TCV, support budget planning for the expansion of the Universal Immunisation Programme (UIP) and assist in designing interventions to enhance financial protection. Together with our previous evidence on the health burden of typhoid fever in India, these findings on the economic burden further illustrate the importance of incorporating TCV into India's immunisation strategy and highlight the economic benefits of reducing both typhoid fever incidence and AMR. As India contributes a considerable share of global typhoid incidence, optimal typhoid fever prevention and control strategies in India would substantially reinforce regional and global initiatives targeting typhoid fever and AMR.

## Introduction

Typhoid fever is a febrile illness caused by *Salmonella enterica* serovar Typhi (*S.* Typhi), which affects multiple organ systems. It is a recognised public health challenge in India because of its high incidence and antimicrobial resistance (AMR).^1^^,^^2^ The population-based, multisite Surveillance for Enteric Fever in India (SEFI) study (2017–2020) showed wide geographic variation in incidence, ranging from 12 to 1622 per 100,000 person-years across Indian settings.^1^ As antimicrobials are the mainstay of treatment, AMR is increasingly recognised as a key barrier to effective management of the illness. Since multidrug resistance (MDR), defined as resistance to ampicillin, chloramphenicol, and trimethoprim–sulfamethoxazole, became common in the 1990s, alternative agents like fluoroquinolones, third-generation cephalosporins, and azithromycin began to be widely used.^3^ The systematic review by Britto et al. reported 66% fluoroquinolone resistance (FQR) during 2011–2015.^4^ In another systematic review and meta-analysis conducted by us to estimate the burden of AMR from 1977 to 2024, we inferred near-zero MDR, 63% resistance for fluoroquinolones, and 3% resistance each for third-generation cephalosporins and azithromycin after 2020.^5^ AMR in *S*. Typhi leads to more hospitalisations and complications, longer duration of illness, and increased cost-of-illness.^6^

The cost-of-illness (COI) evidence for typhoid fever in India is sparse but shows substantial variation by hospitalisation status, sector (public versus private), and facility level. A systematic review identified three COI studies in India, two from the early 2000s and one from 2017 to 2020.^7^ We identified a fourth prospective COI study among non-hospitalised laboratory-confirmed typhoid fever patients conducted in Navi Mumbai between 2018 and 2021 (see Annex 1 for details). Across these four studies, COI ranged from US$22 to US$ 1735 (2022 US$), depending on the setting.^7^, ^8^, ^9^, ^10^, ^11^ All four studies estimated COI in hospitalised patients, ranging from US$ 182 to US$ 976, with a higher cost of US$ 1735 for complications such as intestinal perforation due to typhoid fever in the SEFI study.^8^ Among the three studies reporting non-hospitalised costs, the two early-2000s studies estimated US$ 20 and US$ 114,^9^^,^^10^ whereas the Navi Mumbai study reported US$ 225 (see Annex 1 for details). Two recent studies disaggregated costs by public and private facilities.^8^ The Navi Mumbai study reported a mean cost per case (direct and indirect) of US$ 185 for public facilities and US$ 475 for private facilities (see Annex 1 for study details). SEFI reported a mean direct cost of hospitalisation of US$ 97 in public facilities and US$ 369 in private facilities.^8^ The SEFI study also reported costs by facility size and type. The hospitalisation cost was US$ 204 in smaller and medium health facilities and US$ 597 in large hospitals.^7^^,^^8^ A study conducted in Kolkata, India, estimated that public health facility costs account for 37.5% of total direct and indirect costs.^9^ None of the studies reported separate costs for AMR typhoid fever, and the economic burden due to AMR among typhoid fever patients in India is unknown.

The National Technical Advisory Group on Immunisation in India (NTAGI) recommended Typhoid Conjugate Vaccine (TCV) for inclusion in the Universal Immunisation Programme (UIP) in 2022, although it has not yet been implemented (as of November 2025). The recommendation is to co-administer TCV with the measles-containing vaccine at 9–12 months, with options for catch-up and school-based strategies.^12^ We identified research priorities to support TCV decision-making in India through an evidence assessment and stakeholder survey in 2023.^2^^,^^13^ Our identified priorities included the health and economic burden, typhoid mortality, and AMR tracking. We subsequently updated the AMR systematic review and meta-analysis,^5^ and performed a modelling study estimating India's typhoid burden by age, state, and AMR category in 2023,^14^ demonstrating a high burden of FQR typhoid fever in India.

In this study, we addressed the evidence gap on the economic burden of typhoid fever in India from government and societal perspectives. The analysis is disaggregated by fluoroquinolone resistance (FQR) status, age group, and provincial state-level variations, along with the burden of catastrophic health expenditure.

## Methods

**Fig. 1:**
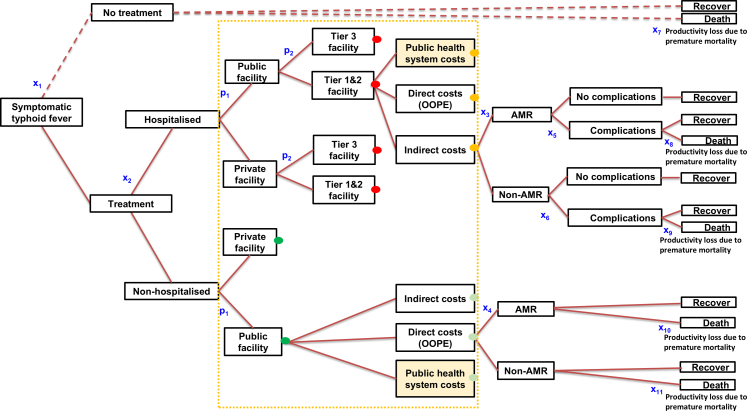
**Decision-tree model for estimating the economic burden of typhoid fever in India**. State transition probabilities are represented in x_1_ to x_11_, p_1_ and p_2_. x_1_ is probability of no treatment among symptomatic typhoid fever; x_2_ is probability of hospitalisation among treated typhoid fever; x_3_ and x_4_ is probability of AMR in hospitalised and non-hospitalised patients; x_5_ and x_6_ is probability of complications in hospitalised AMR and non-AMR patients; x_7_ is probability of death among non-treated symptomatic patients; x_8_ and x_9_ is probability of death among hospitalised AMR and non-AMR with complications; x_10_ is probability of death among non-hospitalised AMR patients; x_11_ is probability of death among non-hospitalised non-AMR patients; p_1_ is probability of typhoid fever treated in public facilities; p_2_ is probability of typhoid fever treated in tier 3 facilities. Each arm in the decision-tree is associated with a cost. Oval nodes in green (non-hospitalised) and red (hospitalised) represent continuing branches that are not shown in the figure. Tier 1 & 2, primary/secondary or small/medium-sized facilities; Tier 3, tertiary hospitals.

We developed a decision-tree model of the treatment pathway for typhoid fever to estimate its economic burden in India (see Fig. 1). The economic burden in our study refers to the total direct and indirect costs associated with typhoid fever in 2023, including public health system costs, out-of-pocket expenditures (OOPE), and lost productivity due to illness and premature death. The treatment pathway in Fig. 1 represented the typhoid fever patients, treatment-seeking, hospitalisations, AMR, clinical complications, and outcomes (survival/deaths),^14^ accounting for direct and indirect costs borne by the government and households across various decision-tree arms. The costing branches included public and private service providers, and by tier: tertiary hospitals (Tier 3) and primary/secondary or small/medium-sized facilities (Tier 1 and Tier 2). We included productivity losses related to premature mortality from typhoid fever as indirect costs. Finally, we summarised the economic burden for 2023, categorised by age, state, and AMR status.

Population data for our analysis were derived from the 2011 Census of India, which projected the population for 2023^15^ (Annex 2). We included data from 34 States and Union Territories (UTs); Telangana was included with Andhra Pradesh, and Ladakh was included with Jammu and Kashmir; Lakshadweep was excluded because typhoid fever incidence data were not available. Age categories were organised into four groups: 6 months-4 years, 5–9 years, 10–14 years, and ≥15 years, matching the age groups in the typhoid fever incidence data from the SEFI project.^1^

We mainly used data from the multisite surveillance study, SEFI project (2017–2020), which is the most recent and nationally representative blood culture-confirmed typhoid fever incidence data from India^1^^,^^16^ and from our systematic review and meta-analysis.^5^ We derived state-wise typhoid fever incidence data by age groups, healthcare utilisation, hospitalisation rate, complications and case fatality rate (CFR) from SEFI data^14^ and presented in Annex 2 & 3. In this analysis, AMR refers specifically to FQR, the dominant resistance pattern in India. The prevalence of FQR in Indian states among hospitalised and non-hospitalised was derived from our systematic review^5^^,^^14^ (Annex 2). Various probabilities for decision-tree nodes (x_1_ to x_11_ in Fig. 1) were calculated both from SEFI and literature reviews^14^ (see Annex 3). The SEFI incidence estimates incorporated adjustments for under-reporting arising from incomplete capture of febrile illnesses at surveillance sites, non-performance of blood culture among eligible patients, and imperfect blood-culture sensitivity.^1^ In addition to these adjustments embedded within the incidence inputs, our decision-tree framework explicitly modelled treatment non-seeking among symptomatic patients (x_1_), thereby incorporating those who did not enter the formal healthcare system.

We estimated costs from both government and societal perspectives. The government perspective included all public health system (provider) expenditures incurred in managing typhoid fever in public-sector facilities. The societal perspective additionally incorporated household OOPE and productivity losses associated with illness and premature mortality.

From a societal perspective, direct costs (Fig. 1) included all direct medical OOPE, such as payments for hospital services, medications, and diagnostics, as well as direct non-medical OOPE, including household transportation and accommodation expenses. Indirect costs refer to the value of lost productivity for patients and caregivers due to time taken away from their usual activities. We estimated productivity losses due to premature mortality separately as the present value of lifetime income lost from typhoid-related deaths among hospitalised, non-hospitalised, and non-treatment-seeking patients, using the human-capital approach (HCA). We conducted a scenario analysis of premature mortality costs using the friction-cost approach (FCA).

Public health system costs reflected public provider expenditures on outpatient visits and hospitalisation for typhoid fever. These costs included staff time, diagnostics, medicines, consumables and overheads. We reported them separately from household costs to maintain a clear distinction between the government and societal perspectives.

The direct and indirect cost estimates for hospitalised were sourced from the published SEFI multicentre cost-of-illness study,^8^ and those for non-hospitalised from a recent prospective cost-of-illness study of laboratory-confirmed typhoid fever conducted in Navi Mumbai (Table 1). A summary of the Navi-Mumbai study, including the sample size and methodology, is presented in Annex 1. Direct costs included medical expenses (consultations, diagnostics, procedures, and medicines) and non-medical expenses (food, transport, lodging, and other out-of-pocket payments). Indirect costs included income loss and payments for replacement labour. In the original studies, the time lost due to productivity losses among patients and caregivers was classified differently: in the SEFI study, it was included under direct costs, whereas in the Navi Mumbai study, it was classified as an indirect cost. Consequently, in our analysis, direct costs for hospitalised patients include productive time losses, while for non-hospitalised, these losses appear under indirect costs. Participants in these typhoid cost-of-illness studies either lacked health insurance or were unable to use it, resulting in no financial contributions from health insurance systems. Neither study estimated the government (provider) costs borne by public health facilities. As there were no provider cost data available, we derived them from the Kolkata typhoid cost-of-illness study,^9^ using the ratio of public-sector to private-sector treatment costs, and applied them to estimate government costs for public facilities (Table 1).

**Table 1:** Model input parameters, distributions and data sources used in the typhoid fever economic burden decision-tree model.

Input parameter	Input value (95% CI)	Probability distribution	Source	Comments
Population	1,388,163,000	No distribution	(15)	Census of India projected data for 2023. State and age-wise population data are available in Annex 1
Epidemiological parameters in the decision-tree model	Variable	Beta	(14)	Annex 2
Incidence of typhoid fever	360 (297–494) per 100,000 PYs	Gamma	(1, 16)	Data from multi-site typhoid surveillance in India. Input is done by state and age-wise and is available in Annex 1.
Direct costs in hospitalised typhoid fever cases in ∗tier 1& 2 hospitals	INR 8292 (3213–17,621)	Lognormal	(8)	Includes direct medical, direct non-medical costs and lost time in INR 2019.
Direct costs in hospitalised typhoid fever cases in ∗tier 3 hospitals	INR 28,237 (15,371–46,649)	Lognormal	(8)	Includes direct medical, direct non-medical costs and lost time in INR 2019.
Indirect costs in hospitalised typhoid fever cases in tier 1& 2 hospitals	INR 4207 (517–18,286)	Lognormal	(8)	Includes income loss and cost of substitute labour in INR 2019.
Indirect costs in hospitalised typhoid fever cases in tier 3 hospitals	INR 11,211 (226–62,847)	Lognormal	(8)	Includes income loss and cost of substitute labour in INR 2019.
Direct costs in non-hospitalised typhoid fever cases	INR 9762 (4488–18,067)	Lognormal	Annex 1	Includes direct medical and direct non-medical costs in INR 2021.
Indirect costs in non-hospitalised typhoid fever cases	INR 5298 (2474–9613)	Lognormal	Annex 1	Includes lost time, income loss and cost of substitute labour in INR 2021.
Cost ratio of typhoid fever versus enteric fever in tier 1& 2 hospitals	1.09 (0.86–1.33)	Lognormal	(8)	Used in estimating the cost of treating typhoid fever cases
Cost ratio of typhoid fever versus enteric fever in tier 3 hospitals	1.00 (0.89–1.12)	Lognormal	(8)	Used in estimating the cost of treating typhoid fever cases
Cost ratio of public versus overall costs in tier 1& 2 hospitals	0.52 (0.39–0.65)	Lognormal	(8)	Used in estimating the cost of treating typhoid fever cases in public facilities
Cost ratio of public versus overall costs in tier 3 hospitals	0.33 (0.28–0.38)	Lognormal	(8)	Used in estimating the cost of treating typhoid fever cases in public facilities
Cost ratio of private versus overall costs in tier 1& 2 hospitals	1.45 (1.10–1.81)	Lognormal	(8)	Used in estimating the cost of treating typhoid fever cases in private facilities
Cost ratio of private versus overall costs in tier 3 hospitals	1.40 (1.22–1.57)	Lognormal	(8)	Used in estimating the cost of treating typhoid fever cases in private facilities
Cost ratio of no complications versus overall costs in tier 1& 2 hospitals	0.85 (0.67–1.02)	Lognormal	(8)	Used in estimating the cost of treating typhoid fever cases with no complications
Cost ratio of no complications versus overall costs in tier 3 hospitals	0.99 (0.88–1.10)	Lognormal	(8)	Used in estimating the cost of treating typhoid fever cases with no complications
Cost ratio of complications/deaths versus overall costs in tier 1& 2 hospitals	2.62 (1.21–4.03)	Lognormal	(8)	Used in estimating the cost of treating typhoid fever cases with complications/deaths
Cost ratio of complications/deaths versus overall costs in tier 3 hospitals	2.42 (0.62–4.23)	Lognormal	(8)	Used in estimating the cost of treating typhoid fever cases with complications/deaths
Cost ratio of antimicrobial- resistant versus antimicrobial-sensitive cases in public health facilities	1.84 (1.53–2.14)	Lognormal	(18)	Used in estimating the cost of treating typhoid fever with antimicrobial resistance
Cost ratio of antimicrobial-resistant versus antimicrobial-sensitive cases in private health facilities	1.12 (0.28–1.96)	Lognormal	(18)	Used in estimating the cost of treating typhoid fever with antimicrobial resistance
Cost ratio of antimicrobial-resistant versus antimicrobial-sensitive cases in all health facilities	1.50 (1.03–1.96)	Lognormal	(18)	Used in estimating the cost of treating typhoid fever with antimicrobial resistance
Cost ratio of public versus overall costs in non-hospitalised cases	0.72 (0.61–0.84)	Lognormal	Annex 1	Used in estimating the cost of treating typhoid fever in public facilities
Cost ratio of OOPE versus overall costs in non-hospitalised cases	0.99 (0.86–1.12)	Lognormal	Annex 1	Used in estimating the cost of treating typhoid fever in private facilities
Cost ratio of public costs versus OOPE in public facilities	0.38 (0.18–0.57)	Lognormal	(9)	Used in estimating the public health (government perspective) cost of treating typhoid fever in public facilities
Probability of FQR in the cost-of-illness study used for data inputs	0.98 (0.96–0.99)	Beta	(1, 8)	Used in adjusting antimicrobial resistance costs
Probability of FQR by Indian states by hospitalisation status (x_3_ & x_4_)	Varied by states (0.38–1.00)	Beta	(5, 14)	Estimated from a systematic review. Each state had specific inputs, presented in Annex 1
Probability of typhoid fever cases treated in public facilities (p_1_)	Varied by states (0.38–0.63)	Beta	(17)	Presented in Annex 1. We assumed a standard deviation equal to 10% of the mean value.
Probability of typhoid fever cases treated in tier 3 facilities (p_2_)	Varied by states (0.18–0.65)	Beta	(17)	Presented in Annex 1. We assumed a standard deviation equal to 10% of the mean value.
Probability of hospitalised cases resulting in catastrophic out-of-pocket expenditures	0.07 (0.00–0.18) 0.17 (0.11–0.23)	Beta	(8)	0.07 in tier 1 and 2 facilities and 0.17 in tier 3 facilities
GDP per capita by Indian states for 2023	Varied by states	None	(19)	Presented in Annex 1
Life expectancy at birth	Varied by states	None	(21)	Presented in Annex 1
United States Dollars and Indian Rupee (INR) Exchange Rate	1 US$ = INR 82.60	None	(22)	Annual average from the official data source
Discount rate	3%	None	(20)	WHO recommendation

CI, confidence interval; FQR, fluoroquinolone resistance; ∗tier 1 & 2, primary/secondary or small/medium sized facilities; ∗tier 3, tertiary hospitals; GDP, gross domestic product; INR, Indian Rupee; PYs, person-years; OOPE, out-of-pocket expenditures.

We extracted the proportion of typhoid fever treated in both public and private, and tertiary hospitals (Tier 3) and primary/secondary/small/medium-sized facilities (Tier 1 & 2) from a government of India report, and compiled at the state level^17^ (Annex 2). We assumed that fluoroquinolone-resistant infections incur higher treatment costs than sensitive infections; a cost ratio of 1.5 (95% CI 1.03–1.96) was applied based on a multi-hospital cohort study^18^ (Table 1). This pooled AMR cost ratio was used as a proxy because typhoid-specific AMR cost estimates were not available in India. As the empirical cost-of-illness data predominantly reflect fluoroquinolone-resistant patients, we derived costs for non-resistant infections by applying the inverse of the AMR cost ratio rather than estimating them from separate empirical observations.

We estimated productivity loss due to premature mortality using the human capital approach, in which the present value of lost future income was calculated as a product of the number of years of life lost and gross domestic product per capita in 2023,^19^ discounted at 3% per year, following WHO guidelines.^20^ We subtracted 2.5, 7.5, 12.5 and 25 years from life expectancy at birth for 2022 in Indian states^21^ to estimate lost productive years for the age groups 6 months-4 years, 5–9 years, 10–14 years, and ≥15 years, respectively.

Finally, we inflated all costs to Indian Rupee (INR) 2023 using the consumer price index,^19^ conducted calculations in INR and converted to US$ 2023 using the Reserve Bank of India annual average exchange rate for the year 2023 (1 US$ = INR 82.60).^22^

### Statistical analysis

All the input parameters used in the decision-tree model were derived from sampling their probability distributions (Table 1, Annex 2). We used the uncertainty interval (UI) for results from probabilistic sensitivity analysis and the confidence interval (CI) for uncertainty reported in source studies. We performed a multivariate probabilistic sensitivity analysis using Monte Carlo simulations, conducting 5000 independent random draws for each parameter identified using Ersatz,^23^ a Microsoft Excel add-in program. Each simulation produced analytical outputs for 5000 iterations (sufficient for convergence), from which we derived the 95% CI, mean, and median. We calculated Spearman's rank correlation coefficients to assess and rank the influence of individual input parameters on model outputs.

Our previous analysis of typhoid fever disease burden showed that assumptions about age-specific hospitalisation patterns influenced which age groups incurred the highest burden.^14^ Therefore, we estimated the age-specific economic burden under two primary scenarios to account for uncertainty in age-specific hospitalisation, complication, and mortality patterns identified in the disease burden model.^14^ In the Primary Scenario, hospitalisations, complications, and deaths were redistributed by age using GBD 2021 patterns. In Primary Scenario B, we retained the age distribution of hospitalisations, complications, and deaths observed in SEFI surveillance data.^14^ Carrying forward these two scenarios ensured that uncertainty in age-specific clinical severity was consistently reflected in age-specific economic outcomes.

In addition, we conducted an alternative scenario analysis to assess uncertainty in the valuation of productivity losses from premature mortality. In an alternative scenario, we used the friction-cost approach to estimate the productivity loss due to premature mortality. The labour market can replace individuals who are absent and restore productivity to its previous levels without experiencing long-term losses.^24^ The time required to replace a deceased individual with a new, fully capable worker is known as the “friction period”.^24^ In our analysis, we accounted for productivity loss only during this friction period. Based on prior studies conducted in India, we adopted a three-month friction period.^25^^,^^26^ It's important to note that the actual productivity loss is often less than the total productive time lost in the labour market. This discrepancy is referred to as labour market elasticity.^24^ We used a labour market elasticity of 0.8, indicating that the estimated productivity loss is 80% of the productive time lost.^25^ We valued the productive time in state-specific gross domestic product per capita in 2023, similar to the human capital approach.^19^

We presented the economic burden of typhoid fever, organised by state, age group, and fluoroquinolone resistance status, from a societal and government perspective. We also categorised results by OOPE (direct and indirect costs), public costs, and productivity loss due to premature mortality, and separated them by hospitalisation status and the number of households with catastrophic expenditures. If a household spent more than 40% of its annual non-subsistence expenditure (capacity to pay) on typhoid treatment, it was categorised as catastrophic expenditure.^8^

### Ethics statement

This study was approved by the London School of Hygiene & Tropical Medicine (LSHTM) Research Ethics Committee (Ref. No 31427, 27 November 2024). This study used publicly available data and did not involve human subjects.

### Role of the funding source

The funders had no role in study design, data collection, data analysis, data interpretation, or writing of the report. The authors had full access to all the data and final responsibility for the decision to submit for publication.

## Results

In 2023, we estimated a total of 4.7 million (95% UI 4.2–5.4) treatment-seeking typhoid fever patients and 0.2 million (0.16–0.22) that did not seek formal healthcare (Annex 4). These patients resulted in 0.7 million (0.5–1.00) hospitalisations and 4.0 million (3.4–4.6) non-hospitalised episodes. Approximately three-quarters of patients were fluoroquinolone-resistant (Annex 4). We estimated a total of 7800 (2600–19,100) premature deaths, including 5300 (2000–11,800) among healthcare seekers and 2500 (1300–4100) among non-seekers. The direct and indirect costs of typhoid fever treatment were estimated at INR 123.0 billion (95% UI 76.7–217.5 billion; US$ 1.5 billion, 95% UI 0.9–2.6) in 2023 (Table 2). In addition, we estimated the productivity loss at INR 42.6 billion (95% UI 15.7–111.1 billion; US$ 515 million, 95% UI 190–1344).

**Table 2:** The economic burden of typhoid fever in India: economic burden of typhoid fever by antimicrobial resistance (AMR), hospitalisation status, public versus private and premature mortality in India for 2023.

Category of costs	AMR associated costs (INR millions; US$ millions) (95% UI)	non-AMR associated costs (INR millions; US$ millions) (95% UI)	Total costs (INR millions; US$ millions) (95% UI)
**Hospitalised cases**			
Direct costs (OOPE) in hospitalised cases	INR 18,043 (7948–49,621); US$ 218 (96–600)	INR 2753 (1053–8087); US$ 33 (12–97)	INR 20,797 (9001–57,708); US$ 251 (108–698)
Public health system (payer's perspective) costs in hospitalised cases	INR 1819 (773–4844); US$ 22 (9–58)	INR 119 (47–317); US$ 1 (0–3)	INR 1939 (820–5161); US$ 23 (9–62)
Total costs in hospitalised cases	INR 19,863 (8721–54,465); US$ 240 (105–659)	INR 2872 (1100–8404); US$ 34 (13–101)	INR 22,736 (9822–62,870); US$ 275 (118–761)
**Non-hospitalised cases**			
Direct costs (OOPE) in non-hospitalised cases	INR 77,237 (41,816–141,161); US$ 935 (506–1708)	INR 11,556 (6163–21,505); US$ 139 (74–260)	INR 88,793 (47,979–162,667); US$ 1074 (580–1969)
Public health system (payer's perspective) costs in non-hospitalised cases	INR 9628 (4354–21,004); US$ 116 (52–254)	INR 1435 (645–3253); US$ 17 (7–39)	INR 11,064 (5000–24,258); US$ 133 (60–293)
Total costs in non-hospitalised cases	INR 86,865 (46,171–162,166); US$ 1051 (558–1963)	INR 12,991 (6809–24,759); US$ 157 (82–299)	INR 99,857 (52,980–186,925); US$ 1208 (641–2263)
**Treatment-seeking cases**			
Total public health system (payer's perspective) costs	INR 11,448 (5127–25,848); US$ 138 (62–312)	INR 1554 (693–3571); US$ 18 (8–43)	INR 13,003 (6624–26,975); US$ 157 (80–326)
Direct costs (OOPE) in public facilities	INR 32,075 (19,039–55,439); US$ 338 (231–672)	INR 4336 (2424–7920); US$ 53 (30–96)	INR 36,281 (22,064–61,293); US$ 439 (267–742)
Direct costs (OOPE) in private facilities	INR 65,935 (37,985–117,760); US$ 798 (460–1426)	INR 10,462 (5846–18,695); US$ 127 (71–226)	INR 76,398 (45,427–133,250); US$ 925 (550–1613)
Total direct costs (OOPE)	INR 95,281 (49,764–190,783); US$ 1153 (602–2309)	INR 14,310 (7216–29,592); US$ 173 (87–358)	INR 109,591 (70,035–190,524); US$ 1326 (847–2306)
Total costs of treatment-seeking	INR 106,729 (54,8920–216,630); US$ 1292 (665–2623)	INR 15,865 (9910–33,163); US$ 192 (96–402)	INR 122,593 (76,659–217,498); US$ 1484 (928–2633)
**Premature deaths**			
Productivity loss due to premature deaths in hospitalised cases	INR 19,373 (7273–42,272); US$ 234 (88–511)	INR 1403 (401–4655); US$ 16 (4–56)	INR 20,776 (7675–46,928); US$ 251 (92–568)
Productivity loss due to premature deaths in non-hospitalised cases	INR 5947 (227–31,361); US$ 72 (2–379)	INR 1395 (52–8587); US$ 16 (0–103)	INR 7342 (279–39,948); US$ 88 (3–483)
Productivity loss due to premature deaths in treatment non-seekers	NA	NA	INR 14,433 (7783–24,215); US$ 174 (94–293)
Total Productivity loss due to premature deaths	INR 25,320 (7501–73,634); US$ 306 (90–891)	INR 2798 (454–13,242); US$ 33 (5–160)	INR 42,552 (15,739–111,093); US$ 515 (190–1344)
**Total societal costs**	INR 132,050 (88,603–224,878); US$ 1598 (1072–2722)	INR 18,663 (11,705–34,562); US$ 225 (141–418)	INR 165,147 (118,240–268,657); US$ 1999 (1431–3252)

AMR, antimicrobial resistance (refers to fluoroquinolone resistance); INR, Indian Rupee; OOPE, out-of-pocket expenditure; US$, United States Dollars.

**Fig. 2:**
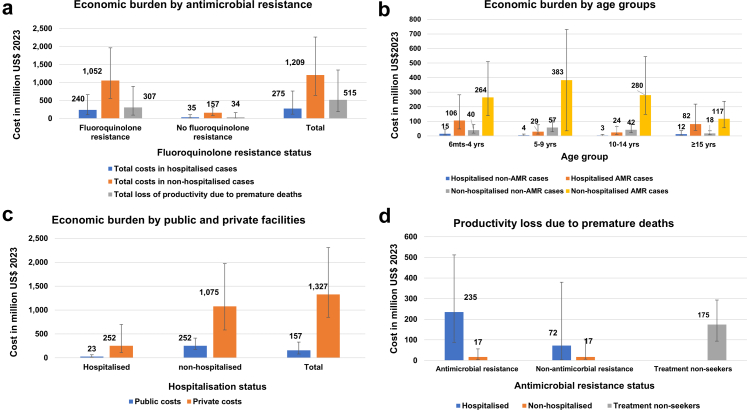
**Economic burden by antimicrobial resistance (a), age groups (b), public versus private costs (c), and productivity loss due to premature deaths (d).** Each bar represents the estimated cost in US$ in millions, with uncertainty intervals represented by error bars for the year 2023.

About 1.8 million (1.5–2.1) non-hospitalised and 0.3 million (0.2–0.4) hospitalised typhoid fever patients were treated in public facilities, costing overall INR 13.0 billion (95% UI 6.6–27.0 billion; US$ 157 million, 95% UI 80–326) to the public health system (government perspective) and INR 36.0 billion (95% UI 22.0–61.3 billion; US$ 439 million, 95% UI 267–742) to individuals (Table 2, Annex 4). About 2.2 million (1.9–2.6) non-hospitalised and 0.4 million (0.3–0.6) hospitalised patients were treated in private facilities, resulting in an economic burden of INR 76.4 billion (95% UI 45.4–133.2 billion; US$ 925 million, 95% UI 550–1613) for affected individuals and their families. Non-hospitalised patients were 5.5 times more common and accounted for 4.4 times the economic burden relative to hospitalised (Fig. 2). The parameters driving these results are presented in probabilistic sensitivity analysis in Annex 5.

### Economic burden due to FQR and by age group

The FQR was associated with an economic burden of INR 107.0 billion (95% UI 54.9–216.7 billion; US$ 1.3 billion, 95% UI 0.7–2.6), constituting 87% of the typhoid fever economic burden in addition to INR 25.3 billion (95% UI 7.5–73.6 billion; US$ 306 million, 95% UI 90–891) economic burden resulting from the lost productivity due to premature deaths (Table 2, Fig. 2). The 5–9-year age group had the highest economic burden in both primary scenarios (Table 2 and Annex 6), and those under 10 constituted more than 60% of the overall economic burden.

### Economic burden from the government perspective, individuals and families

The OOPE constituted INR 109.6 billion (95% UI 70.0–190.5 billion; US$ 1.3 billion, 95% UI 0.9–2.3), and public health cost was about 9% of treatment costs, constituting about INR 13.0 billion (95% UI 6.6–27.0 billion; US$ 157 million, 95% UI 80–326) (Table 2, Fig. 2).

The loss of productivity due to premature mortality from typhoid fever was INR 42.5 billion (95% UI 15.7–111.1 billion; US$ 515 million, 95% UI 190–1344), representing 26% of the total economic burden. About 49% productivity due to premature deaths occurred in hospitalised typhoid fever patients, and 34% occurred outside the health system (Fig. 2).

We estimated 70,000 (37,000–157,000) families with typhoid fever patients in India experienced catastrophic expenditures, which account for 9.6% of all hospitalisations.

**Fig. 3:**
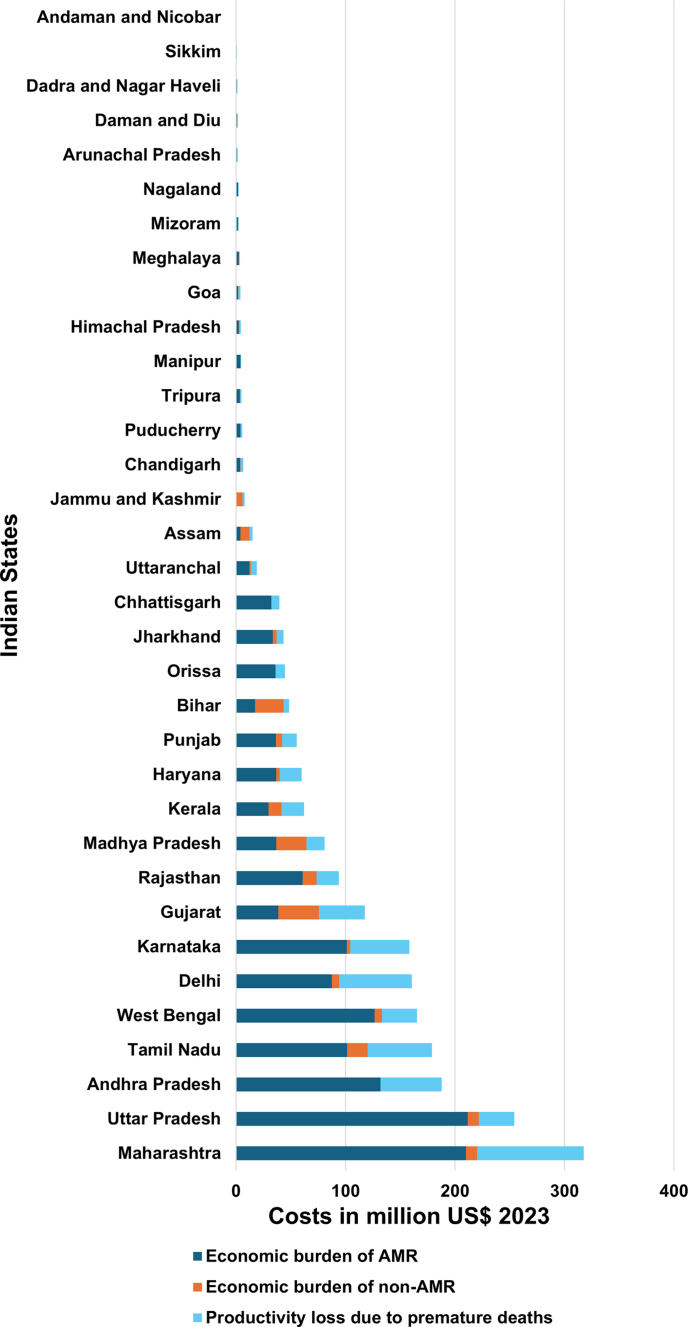
**Cost-of-illness per unit by Indian states**. The graph shows unit costs for hospitalised antimicrobial resistance (AMR), hospitalised non-AMR, non-hospitalised AMR and non-hospitalised non-AMR typhoid fever with uncertainty intervals for Indian states in US$ 2023.

**Fig. 4:**
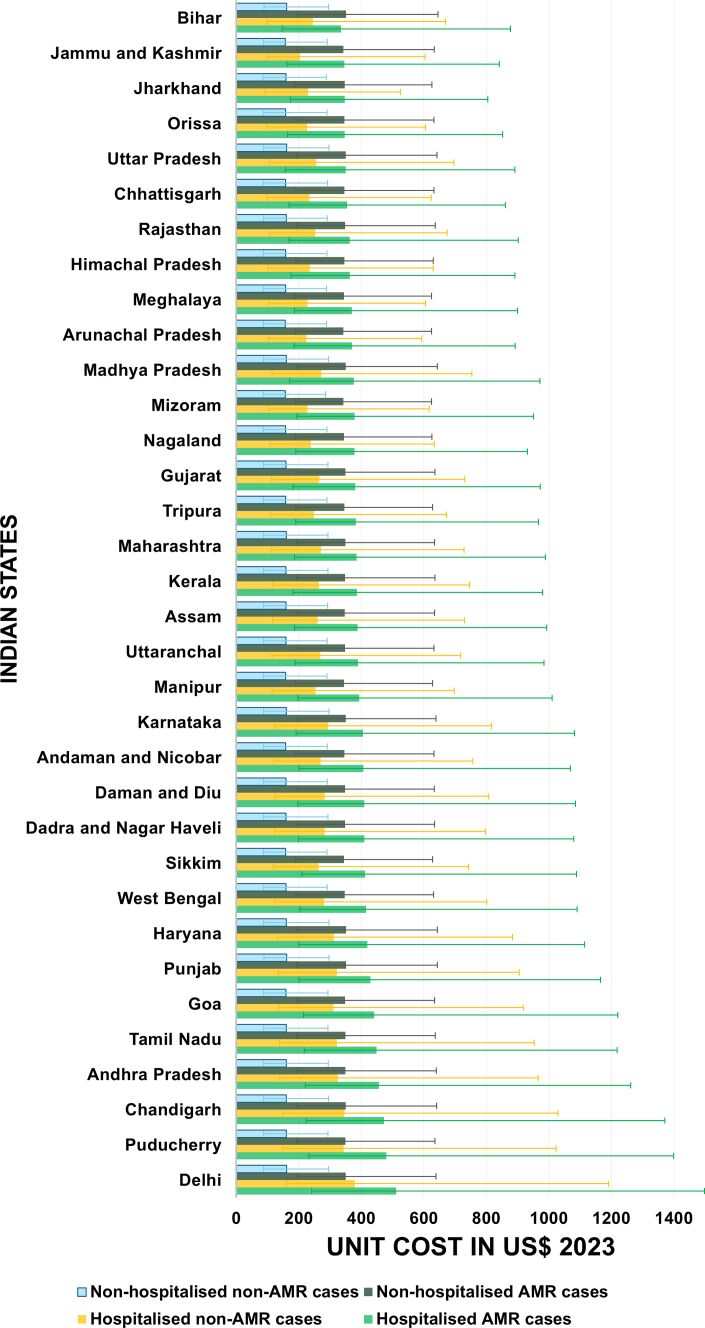
**Economic burden of typhoid fever by Indian states in US$ for 2023**. The graph illustrates the economic burden of antimicrobial resistance (AMR) and non-AMR costs, as well as productivity losses from premature deaths from typhoid fever in Indian states in 2023, in US$ 2023. State-level variation in unit costs is observed for hospitalised patients due to differences in facility mix and care pathways, whereas unit costs for non-hospitalised patients are applied uniformly across states based on available cost data.

The unit cost of a typhoid fever patient was INR 26,514 (95% UI 16,685–44,851; US$ 321, 95% UI 202–543), for hospitalised patient was INR 31,057 (95% UI 16,602–77,230; US$ 376, 95% UI 201–935) and non-hospitalised was INR 24,945 (95% UI 14,372–44,686; US$ 302, 95% UI 174–541). The unit cost for FQR in hospitalised and non-hospitalised typhoid fever patient was INR 33,082 (95% UI 15,951–86,403; US$ 401, 95% UI 193–1046) and INR 22,232 (95% UI 9543–60,553; US$ 269, 95% UI 116–733), respectively. The unit cost for non-FQR hospitalised and non-hospitalised typhoid fever patient was INR 28,553 (95% UI 15,866–52,621; US$ 346, 95% UI 192–637), and INR 13,157 (95% UI 7204–24,316; US$ 159, 95% UI 87–294), respectively. The state-wise unit cost per typhoid fever patient, by AMR and hospitalisation status, were provided in Figs. 3 and 4.

The five provincial states of India with the highest economic burden were Maharashtra, Uttar Pradesh, Andhra Pradesh (including Telangana), Tamil Nadu, and West Bengal, which accounted for 51% of India's overall economic burden.

In the alternative scenario of using the friction cost approach instead of the human capital approach, the productivity loss due to premature mortality was reduced by 99.8% to INR 86.5 million (95% UI 40.1–178.8; US$ 1.0 million, 95% UI 0.5–2.2).

## Discussion

We estimated the economic burden of typhoid fever in India in 2023, accounting for fluoroquinolone resistance, age group of patients, and provincial Indian states, from both government and societal perspectives. We inferred a significant economic burden of typhoid fever at INR 123 billion (US$ 1.5 billion), driven by FQR and concentrated among children less than 10 years of age and in high-incidence Indian states. Households bore 91% of the economic burden, resulting in catastrophic hospitalisation costs, highlighting the financial challenges associated with typhoid fever in India. This economic burden increases further when we add the productivity loss of INR 43 billion (US$ 0.5 billion) from premature mortality, from a societal perspective. While uncertainty around the absolute national cost estimate is high and largely driven by non-hospitalised costs, results from both primary age-specific scenarios show a consistent distribution of economic burden across age groups, states, and FQR.

Our study made three main contributions. First, the study presented national estimates of the economic burden of typhoid fever in India by integrating disease burden, AMR, and costs in a single framework. Second, we quantified the economic consequences of FQR and showed that resistant infections accounted for the majority of national costs, a finding not previously demonstrated at scale. Third, we identified where and among whom the economic burden is concentrated.

Our findings show that FQR accounts for 87% of the total economic burden, aligning with the 82% prevalence of resistance we identified in our systematic review and the 74% among overall national burden in our another study.^5^^,^^14^ We note that most economic costs in FQR typhoid fever stem from their high prevalence; only a smaller share of this burden is directly attributable to resistance itself. The cost-of-illness associated with FQR was substantially higher in both hospitalised and non-hospitalised patients. The high financial burden and catastrophic expenditure among hospitalised typhoid fever patients show the need for better financial risk protection. Evidence from earlier studies, which report 18–27% distress financing through borrowing or asset sales, highlights the persistent economic vulnerability of families affected by typhoid fever.^8^

The distribution of the economic burden across Maharashtra, Uttar Pradesh, Andhra Pradesh (including Telangana), Tamil Nadu, and West Bengal, which together account for 51% of national costs, suggests that state-level implementation strategies for prevention and control will be critical. Children under 10 years of age, especially those aged 5–9, incurred the highest economic burden from typhoid fever. Together with children aged 6 months to 4 years, these groups accounted for over half the total burden, supporting age-prioritised TCV introduction strategies. From the government's perspective, public expenditure accounts for only 9% of total treatment costs, suggesting that government fiscal savings from TCV may be modest. However, from a societal perspective, TCV can significantly reduce household OOPE, catastrophic expenditures, and productivity losses due to premature mortality. Previous cost-effectiveness studies from a societal perspective have shown good value for money, particularly for age-targeted and high-population urban strategies.^27^^,^^28^

When compared with the other two major febrile illnesses in India, the economic burden of typhoid fever is lower than that of dengue (estimated at US$ 4.1 billion in 2016; approximately US$ 5.7 billion in 2023) and malaria (US$ 1.9 billion in 2012; roughly US$ 3.8 billion in 2023).^29^^,^^30^ Differences in disease burden and clinical complications, as well as in study methods, and macro-costing for dengue and malaria versus empirical micro-costing in our analysis, limit direct comparability. Nonetheless, unlike dengue and malaria, typhoid has four licensed TCVs available in India and is already recommended by NTAGI (National Technical Advisory Group on Immunisation), making typhoid prevention actionable in the near future.^2^^,^^12^

Our study has limitations. The 2023 economic burden estimates drew on epidemiological parameters from 2017 to 2020, the most recent nationally representative surveillance data available, while demographic inputs, costs, and AMR patterns were updated to 2023. This approach assumes no major structural change in transmission or care-seeking patterns. We conducted probabilistic sensitivity analyses to reflect parameter uncertainty, assuming that the parameter distributions are appropriate for the population modelled. Some inputs were drawn from studies conducted at different times and in different settings, and the corresponding uncertainty intervals may not fully reflect structural or contextual variation at the national level.

From a costing perspective, we underestimated costs from the government's perspective because the cost-of-illness data we used in our analysis predate the expansion of India's social insurance system, Ayushman Bharat-Pradhan Mantri Jan Arogya Yojana (AB-PMJAY).^31^ This AB-PMJAY has the potential to shift private hospitalisation costs to public insurance, reducing OOPE. In addition, public provider costs were estimated using a historical public-to-private cost ratio, which may further underestimate current government expenditures given changes in health financing over time. We applied a single national AMR cost ratio for public and private facilities because state-level cost data were unavailable. Therefore, our AMR cost estimates may not fully reflect geographic variation in treatment costs driven by differences in prices, clinical practices, and healthcare delivery systems.

The cost-of-illness data for non-hospitalised patients were collected in Navi Mumbai, an urban area, potentially leading to an overestimation of national averages. Also, cost-of-illness studies often oversample large hospitals, leading to higher cost estimates. We used the same unit cost inputs across all age groups. But the treatment costs may vary by age due to factors such as disease severity, length of hospital stay, and level of care required. Therefore, the age-specific economic estimates may not fully reflect differences in costs across age groups. Furthermore, the cost per episode of infectious diseases estimated from India's National Sample Survey (2017–18) self-reported household expenditure data indicates lower OOPE for other infectious disease episodes.^32^ However, this could be due to differences in study design and disease characteristics.

There is also substantial uncertainty in the productivity loss due to premature mortality, as shown by the 99.8% reduction observed under the friction-cost approach compared to the human capital approach. Because these two estimates rest on different assumptions, the productivity loss from premature mortality should be interpreted cautiously and in context. We captured indirect costs for paediatric patients through caregiver productivity loss and productivity loss due to premature mortality, while loss of schooling or children's own productive time was not monetised. In addition, we did not assign OOPE or morbidity-related productivity losses to patients who did not seek formal healthcare, which may result in conservative estimates of the total economic burden.

Finally, this analysis was limited to illness-related costs and did not include surveillance, programme implementation, vaccination delivery, or broader macroeconomic effects such as impacts on tourism. Therefore, the total societal impact of typhoid fever may be underestimated.

In summary, typhoid fever contributes to a high socioeconomic burden among Indian households. The economic burden is particularly concentrated among children less than 10 years of age and in high-incidence Indian states, driven by widespread resistance to fluoroquinolones. Implementing TCV and AMR control measures, such as antibiotic stewardship, is essential to reduce both the health and economic consequences of typhoid fever. Strengthening diagnostic capacity and ensuring effective therapeutic management are also important complementary measures to reduce transmission and chronic carrier status of the infection. Additionally, strengthening financial risk protection measures, particularly through existing initiatives such as AB-PMJAY, could further alleviate household vulnerability. As a significant proportion of the global burden of typhoid fever is attributed to India, effective and practical control measures in India would greatly enhance regional and global strategies against enteric fever and AMR.

## Contributors

Conceptualisation: VVM, WJE, AC, KA, VM; Data curation: VVM; Formal analysis: VVM; Funding acquisition: VVM, WJE, KA; Investigation: VVM; Methodology: VVM, WJE, AC, KA, VM; Project administration: VVM; Resources: VVM, JJ, AR, HHF; Software: VVM; Supervision: WJE, BGD, AC, KA; Validation: VVM, KA, VM; Visualisation: VVM; Writing—original draft: VVM; Writing—review & editing: JJ, AR, HHF, VM, RH, BGD, WJE, AC, KA; WJE, AC, and KA contributed equally. All authors have approved the final version. VVM, KA and VM have accessed and verified the data, and all authors were responsible for the decision to submit the manuscript.

## Data sharing statement

Data available within the article or its Supplementary Materials.

## Declaration of interests

The contents of this article are solely the responsibility of the authors and do not represent the official views of their affiliated organisations. Where authors are identified as personnel of the WHO, the authors alone are responsible for the views expressed in this article, and they do not necessarily represent the decisions, policy, or views of the WHO. The authors declare no other competing interests.

## Appendix A Supplementary data

Annex
