# Supplementary material for: Economic burden of typhoid fever by antimicrobial resistance in India: a modelling study 2023
Source: Lancet Reg Health Southeast Asia. 2026 Mar 20;47:100748. doi: 10.1016/j.lansea.2026.100748 (PMC13018988; doi:10.1016/j.lansea.2026.100748)
Supplement: Annex [file mmc1.pdf]

Economic burden of typhoid fever by antimicrobial resistance in India: a modelling study 2023

Vijayalaxmi V Mogasale[1,2,3], Jacob John[4], Arindam Ray[5], Habib Hasan Farooqui[6], Vittal Mogasale[7], Raymond Hutubessy[8], Bhim Gopal Dhoubhadel[2,3,†], W John Edmunds[1,2,3,†], Andrew Clark[9,†], Kaja Abbas[1,2,3,10,11,†]*

[1]*Department of Infectious Disease Epidemiology and Dynamics, London School of Hygiene & Tropical Medicine, London, UK*

[2]*School of Tropical Medicine and Global Health, Nagasaki University, Nagasaki, Japan*

[3]*Institute of Tropical Medicine, Nagasaki University, Nagasaki, Japan*

[4]*Department of Community Health, Christian Medical College, Vellore, India*

[5]*Department of Infectious Disease & Vaccine Delivery, Gates Foundation, New Delhi, India*

[6]*College of Medicine, Qatar University, Doha, Qatar*

[7]*Graduate School of Public Health, Yonsei University, Seoul, Republic of Korea (Current affiliation: Health Financing and Economics Department, World Health Organisation, Geneva, Switzerland)*

[8] *Department of Performance, Finance and Delivery, World Health Organisation, Geneva, Switzerland*

[9]*Department of Health Services Research and Policy, London School of Hygiene & Tropical Medicine, London, UK*

[10]*Public Health Foundation of India, New Delhi, India*

[11]*National Institute of Infectious Diseases, Japan Institute for Health Security, Tokyo, Japan*

†Share senior authorship

*Correspondence: Vijayalaxmi V Mogasale, Department of Infectious Disease Epidemiology and Dynamics, London School of Hygiene & Tropical Medicine, London, UK. Email: vijayalaxmi.mogasale@lshtm.ac.uk; vijayalaxmimogasale@gmail.com

Annex

Annex 1: Reported study methodology and summary of two cost-of-illness studies in India

**Parameter**	**Multi-state, multi-site SEFI study** (1)	**Multi-site Navi-Mumbai study**
Study population	Hospitalised, blood culture–confirmed enteric fever (typhoid fever and paratyphoid fever); all age groups	Hospitalised and non-hospitalised, blood culture–confirmed typhoid fever; under 16 years
Study period	November 2017 to March 2020	October 2018 to March 2021
Study settings	14 sites: 5 rural and 1 urban small hospitals (tier 2), and 8 tertiary hospitals (tier 2); across India	6 sites: small and large hospitals across the Navi-Mumbai district of India
Sample size	274 (tier 2), 891 (tier 3), 100 ileal perforations	37 hospitalised and 28 non-hospitalised
Perspective	Household (patient + caregiver)	Household (patient + caregiver)
Health-system/provider costs	Not estimated	Not estimated
Direct medical costs	Consultation fees, diagnostics, procedures, and medicines	Consultation fees, diagnostics, procedures, and medicines
Direct non-medical costs	Food, transport, lodging, other out-of-pocket payments and productive time lost by patients and caregivers	Food, transport, lodging, and other out-of-pocket payments
Indirect costs	Income loss and substitute labour costs (alternative productivity arrangements) for patients and caregivers. The loss of schooling or children's own productive time was not monetised.	Productive time lost, income loss and substitute labour costs by patients and caregivers. The loss of schooling or children's own productive time was not monetised (except in the GDP per capita method, see below)
Indirect cost approach	Human-capital approach	Human-capital approach
Treatment of productivity loss	Self-reported time loss/income loss/ costs	Self-reported time loss was valued in 3 methods: 1) minimum wage per day for those reporting income loss, 2) self-reported income loss, and 3) GDP per capita per day

Recall period	Onset to 28 days post-discharge	Up to 4 prospective follow-up interviews; up to 90 days if required
Average direct cost: hospitalised case	INR 8,292 (US$ 119) in tier 2; INR 28,238 (US$ 406)in tier 3; for enteric fever cases; presented in INR(US$) 2019	INR 20,209 (US$ 283) for typhoid fever cases; presented in INR(US$) 2021 in minimum wage per day method
Average indirect cost: hospitalised case	INR 4,708 (US$ 68) in tier 2; INR 11,211 (US$ 161) in tier 3; for enteric fever cases; presented in INR(US$) 2019	INR 5,530 (US$ 78) for typhoid fever cases; presented in INR(US$) 2021 in minimum wage per day method
Average direct cost: non-hospitalised case	Not available	IN 9,762 (US$ 137) for typhoid fever cases; presented in INR(US$) 2021 in minimum wage per day method
Average indirect costs: non-hospitalised case	Not available	INR 5,298 (US$ 74) for typhoid fever cases; presented in INR(US$) 2021 in minimum wage per day method
Total productivity loss days for patients and caregivers	Average 8.6 days in tier 2 and average 11.0 days in tier 3; hospitalised case	Average 26.9 days for hospitalised and non-hospitalised cases
Main cost driver	Drugs and consumables (tier-2); hospital charges (tier-3)	Treatment costs in private facilities

Annex 2: State-wise population, typhoid fever incidence, antimicrobial resistance, hospital utilisation, per capita income and life expectancy

	State/union territory*	Total population	typhoid fever incidence per 100,000 PYs (Mean (95%UI)	Probability of FQR in hospitalised cases, x_3 (95%UI)	Probability of FQR in non–hospitalised cases, x_4 (95%UI)	Probability of hospitalisation in public facilities, p_1 (95%UI)	Probability of hospitalisation in tertiary facilities, p_2 (95%UI)	GDP per capita in INR 2023	Life expectancy at birth for the year 2022
1	Andaman and Nicobar	403,000	390 (321–534)	1.00 (0.96–1.00)	0.95 (0.76–1.00)	0.52 (0.42–0.62)	0.42 (0.34–0.50)	258,151	71.26
2	Andhra Pradesh	91,246,000	204 (175–286)	0.86 (0.80–0.94)	0.77 (0.64–0.75)	0.44 (0.35–0.53)	0.52 (0.42–0.62)	266,202	68.19
3	Arunachal Pradesh	1,562,000	166 (144–235)	0.95 (0.88–1.00)	0.84 (0.70–0.86)	0.61 (0.49–0.73)	0.35 (0.28–0.42)	199,992	71.65
4	Assam	35,713,000	162 (140–230)	0.73 (0.67–0.79)	0.64 (0.54–0.63)	0.51 (0.41–0.61)	0.36 (0.29–0.43)	119,308	68.42
5	Bihar	126,756,000	305 (253–421)	0.43 (0.39–0.46)	0.38 (0.32–0.37)	0.38 (0.31–0.45)	0.18 (0.14–0.22)	53,478	65.65
6	Chandigarh	1,231,000	1245 (963–1702)	0.98 (0.91–1.00)	0.87 (0.73–0.93)	0.41 (0.33–0.49)	0.55 (0.44–0.66)	399,654	70.27
7	Chhattisgarh	30,180,000	400 (339–540)	0.86 (0.79–0.93)	0.76 (0.63–0.74)	0.52 (0.42–0.62)	0.27 (0.22–0.32)	137,329	67.13
8	Dadra and Nagar Haveli	699,000	457 (374–624)	0.68 (0.63–0.74)	0.61 (0.51–0.59)	0.48 (0.39–0.57)	0.41 (0.33–0.49)	169,496	68.8
9	Daman and Diu	564,000	393 (326–536)	0.68 (0.63–0.74)	0.61 (0.51–0.59)	0.48 (0.39–0.57)	0.41 (0.33–0.49)	169,496	71.24
10	Delhi	21,359,000	149 (130–213)	0.92 (0.86–1.00)	0.82 (0.68–0.81)	0.40 (0.32–0.48)	0.65 (0.52–0.78)	430,120	69.4
11	Goa	1,575,000	249 (210–346)	1.00 (0.95–1.00)	0.94 (0.76–1.00)	0.47 (0.38–0.56)	0.50 (0.40–0.60)	492,648	72.51
12	Gujarat	71,507,000	298 (248–411)	0.81 (0.75–0.88)	0.72 (0.60–0.71)	0.46 (0.37–0.55)	0.32 (0.26–0.38)	272,451	68.44
13	Haryana	30,209,000	441 (362–602)	0.98 (0.91–1.00)	0.87 (0.73–0.93)	0.39 (0.31–0.47)	0.40 (0.32–0.48)	296,592	68.03
14	Himachal Pradesh	7,468,000	429 (356–582)	0.93 (0.86–1.00)	0.83 (0.69–0.83)	0.54 (0.43–0.65)	0.30 (0.24–0.36)	218,788	69.73
15	Jammu and Kashmir	13,903,000	305 (255–419)	0.84 (0.78–0.91)	0.74 (0.62–0.73)	0.63 (0.51–0.75)	0.28 (0.23–0.33)	130,492	71.75
16	Jharkhand	39,466,000	515 (418–703)	0.54 (0.50–0.59)	0.48 (0.40–0.47)	0.50 (0.40–0.60)	0.24 (0.19–0.29)	96,449	66.84
17	Karnataka	67,692,000	342 (283–470)	0.82 (0.76–0.90)	0.73 (0.61–0.72)	0.42 (0.34–0.50)	0.37 (0.30–0.44)	304,474	69.88
18	Kerala	35,776,000	254 (211–354)	1.00 (1.00–1.00)	1.00 (0.84–1.00)	0.48 (0.39–0.57)	0.34 (0.27–0.41)	252,662	73.43
19	Madhya Pradesh	86,579,000	444 (359–609)	0.67 (0.62–0.73)	0.60 (0.50–0.58)	0.41 (0.33–0.49)	0.29 (0.23–0.35)	132,010	66.46
20	Maharashtra	126,385,000	264 (224–365)	0.78 (0.72–0.85)	0.69 (0.58–0.68)	0.45 (0.36–0.54)	0.33 (0.27–0.39)	252,389	70.12
21	Manipur	3,223,000	224 (190–313)	0.67 (0.62–0.73)	0.59 (0.49–0.58)	0.56 (0.45–0.67)	0.40 (0.32–0.48)	111,853	69.96
22	Meghalaya	3,349,000	427 (353–580)	0.95 (0.88–1.00)	0.84 (0.70–0.86)	0.59 (0.47–0.71)	0.34 (0.27–0.41)	123,896	68.34
23	Mizoram	1,238,000	307 (256–424)	1.00 (1.00–1.00)	1.00 (0.84–1.00)	0.62 (0.50–0.74)	0.38 (0.31–0.45)	215,144	69.82
24	Nagaland	2,233,000	199 (174–276)	1.00 (0.95–1.00)	0.94 (0.76–1.00)	0.58 (0.47–0.69)	0.36 (0.29–0.43)	145,537	69.75
25	Odisha	46,276,000	494 (407–669)	0.60 (0.56–0.65)	0.53 (0.44–0.52)	0.53 (0.43–0.63)	0.25 (0.20–0.30)	143,768	67.54

26	Puducherry	1,646,000	285 (237–394)	0.98 (0.91–1.00)	0.87 (0.73–0.93)	0.45 (0.36–0.54)	0.60 (0.48–0.72)	245,180	73
27	Punjab	30,730,000	282 (235–390)	1.00 (0.94–1.00)	0.90 (0.75–0.98)	0.38 (0.31–0.45)	0.42 (0.34–0.50)	181,678	68.6
28	Rajasthan	81,025,000	360 (302–490)	0.77 (0.71–0.83)	0.68 (0.57–0.67)	0.46 (0.37–0.55)	0.27 (0.22–0.32)	150,653	68.1
29	Sikkim	689,000	395 (323–453)	1.00 (0.95–1.00)	0.93 (0.76–1.00)	0.57 (0.46–0.68)	0.46 (0.37–0.55)	520,466	71.1
30	Tamil Nadu	76,860,000	360 (297–494)	0.79 (0.73–0.86)	0.70 (0.58–0.69)	0.44 (0.35–0.53)	0.50 (0.40–0.60)	277,802	70.54
31	Tripura	4,147,000	390 (321–534)	0.80 (0.74–0.87)	0.71 (0.59–0.70)	0.55 (0.44–0.66)	0.36 (0.29–0.43)	157,364	66.94
32	Uttar Pradesh	235,687,000	204 (175–286)	0.73 (0.68–0.79)	0.65 (0.54–0.63)	0.39 (0.31–0.47)	0.22 (0.18–0.26)	84,126	65.46
33	Uttaranchal	11,637,000	166 (144–235)	0.89 (0.82–0.96)	0.78 (0.66–0.77)	0.48 (0.39–0.57)	0.35 (0.28–0.42)	230,994	67.72
34	West Bengal	99,084,000	162 (140–230)	0.72 (0.67–0.78)	0.64 (0.53–0.62)	0.51 (0.41–0.61)	0.44 (0.35–0.53)	139,442	69.7
	**Total/average**	**1,388,163,000**	**305 (253–421)**	**0.91 (0.84–0.99)**	**0.80 (0.67–0.79)**			**169,496**	**71.7**

*We included data for 34 States and Union Territories; Telangana was included with Andhra Pradesh, Ladakh was included with Jammu and Kashmir, and Lakshadweep was excluded because there was no available incidence data for typhoid fever.

Annex 3: Epidemiological input parameters, distribution and data sources used in the typhoid fever decision tree model (2)

	Parameter name	Value	Type of distribution	Source	Comments
1	Population	1,388,163,000	No distribution	(3)	Census of India projected data for 2023. State and age-wise population data are available in Annex 1
2	Incidence of typhoid fever (base-case)	Overall, 360 (297–494) per 100,000 PYs	gamma	(4, 5)	Data from multi-site typhoid surveillance in India. Input is done by state and age-wise. Data is available in Annex 1& 2
3	Probability of incidence by age groups	Input by age group	beta	(4)	Data from multi-site typhoid surveillance in India. Input by age group: 6 months-4 years = 0.25 (0.20–0.33); 5-9 years = 0.36 (0.31–0.43); 10-14 years = 0.27(0.22–0.33); ≥15 years 0.11(0.08–0.16)
4	Probability of treatment non-seeking(x_1)	0.038 (95% CI: 0.035–0.042)	beta	(4, 6)	Healthcare utilisation survey conducted under SEFI India
5	Probability of hospitalisation (x_2)	0.155 (0.118–0.204)	beta	(2, 4, 7)	Recalculated from SEFI data. We redistributed hospitalisation probability by age-groups (6 months-4 years = 44%, 5-9 years =12%, 10-14 years =10%, and ≥15 years = 34%) based on the global burden of typhoid fever study 2021.
6	Probability of AMR in hospitalised cases (x_3)	0.798 (0.785–0.810)	beta	(8)	Estimated from the systematic review
7	Probability of AMR in non-hospitalised cases(x_4)	0.706 (0.627–0.786)	beta	(8)	Estimated from the systematic review. Proportion of AMR in hospitalised cases/ non-hospitalised cases= 0.89
8	Overall probability of FQR by Indian states	Ranged from 0.38 to 1	beta	(8)	Estimated from a systematic review. Each state had specific inputs, see Annex 3. This was used to estimate x_3 and x_4
9	Probability of Multidrug Resistance (MDR)	0.02 (0.01–0.04)	beta	(8)	Estimated from a systematic review.
10	Probability of third-generation cephalosporins resistance (3GCR)	0.03 (0.01–0.04)	beta	(8)	Estimated from a systematic review
11	Probability of azithromycin resistance (AZR)	0.03 (0.02–0.05)	beta	(8)	Estimated from a systematic review
12	Probability of complications in hospitalised AMR cases (x_5)	0.040 (0.028–0.052)	beta	(4)	Recalculated from SEFI data
13	Odds ratio of complications in non-AMR vs AMR cases	0.496 (0.394–0.625)	lognormal	(9)	Literature review conducted by WHO typhoid advisory group for SAGE meeting
14	Probability of complications in hospitalised non-AMR cases (x_6)	0.0197(0.0196–0.0199)	beta	(4, 9)	Calculated from x_5 using the odds ratio of complications in non-AMR vs AMR cases. Distribution depended on the distribution of x_5 and the odds ratio

15	Probability of deaths (CFR) among healthcare non-seekers (x_7)	0.013 (0.006–0.020)	beta	(4, 10)	Recalculated from SEFI data
16	Probability of deaths (CFR) among hospitalised AMR cases with complications (x_8)	0.146 (0.038–0.255)	beta	(4, 10)	Recalculated from SEFI data
17	Odds ratio of deaths in non-AMR vs AMR cases	0.850 (0.500–1.450)	lognormal	(9)	Literature review conducted by the WHO Typhoid Advisory Group for the SAGE meeting.
18	Probability of deaths (CFR) among hospitalised non-AMR cases with complications (x_9)	0.124 (0.032–0.229)	beta	(4, 9)	Calculated from x_8 using the odds ratio of deaths in non-AMR vs AMR cases. Distribution depended on the distribution of x_8 and the odds ratio.
19	Probability of deaths (CFR) among non-hospitalised AMR cases (x_{10})	0.0005(0.00–0.001)	beta	(4, 10)	Estimated by SEFI (FQR=100%).
20	Probability of deaths (CFR) among non-hospitalised non-AMR cases (x_{11})	0.0004(0.00–0.0005)	beta	(4, 9)	Calculated from x_{10} using the odds ratio of deaths in non-AMR vs AMR cases. Distribution depended on the distribution of x_{10} and the odds ratio.

3GCR = third-generation cephalosporin resistance; AMR=antimicrobial resistance; AZR = azithromycin resistance; CFR= Case fatality Rate; FQR= fluoroquinolone resistance; GBD= Global Burden of Disease; IHME= The Institute for Health Metrics and Evaluation; PYs = person years; SAGE= The Strategic Advisory Group of Experts on Immunization; SEFI= Surveillance for Enteric Fever in India; MDR = multi-drug resistance; WHO= World Health Organisation.

Annex 4: Number of typhoid fever cases and deaths in public and private healthcare systems in India (2)

Category	AMR typhoid fever cases (95% UI)	Non/unknown-AMR typhoid fever (95% UI)	Total typhoid fever (95% UI)
**Hospitalised cases**			
Hospitalised typhoid fever cases in private facilities	335,047 (242,696–444,039)	72,589 (46,500–110,223)	407,637 (289,197–554,262)
Hospitalised typhoid fever cases in public facilities	265,530 (192,060–355,325)	57,783 (37,207–87,704)	323,314 (229,267–443,029)
Total hospitalised typhoid fever cases	600,578 (438,842–795,453)	130,373 (84,478–197,374)	730,951 (523,321–992,828)
Total complications in hospitalised typhoid fever cases	23,622 (14,864–35,720)	2,469 (1,209–4,857)	26,174 (16,556–39,541)
**Non- Hospitalised cases**			
Non-hospitalised typhoid fever cases in private facilities	1,683,177 (1,451,539–1,950,074)	548,334 (453,285–655,663)	2,231,512 (1,904,824–2,605,738)
Non-hospitalised typhoid fever cases in public facilities	1,333,995 (1,138,084–1,557,557)	435,017 (355,434–520,555)	1,769,013 (1,493,519–2,078,112)
Total non-hospitalised typhoid fever cases	3,017,173 (2,622,437–3,465,268)	983,352 (817,959–1,161,821)	4,000,525 (3,440,397–4,627,090)
**Treatment-seeking cases**			
Total cases in the private health system	2,018,225 (1,694,235–2,394,113)	620,924 (499,786–765,887)	2,639,149 (2,194,021–3,160,000)
Total cases in the public health system	1,599,526 (1,330,144–1,912,882)	492,801 (392,642–608,259)	2,092,328 (1,722,786–2,521,142)
Total treatment-seeking cases	3,617,751 (3,024,380–4,306,996)	1,113,725 (892,428–1,374,146)	4,731,477 (4,190,640–5,350,210)
**Treatment-non-seeking cases**			
Total treatment non-seeking cases	NA	NA	188,706 (161,734–219,864)
**Premature deaths**			
Deaths in hospitalised cases	3,580 (1,177–7,126)	299 (77–859)	3,879 (1,254–7,986)
Deaths in non-hospitalised cases	1,104(36–5,387)	287 (9–1,638)	1,390 (45–7,026)
Total deaths in treatment seekers	4,684 (1,213–12,514)	586 (86–2,498)	5,269 (2,008–11,800)
Deaths in treatment non-seekers	NA	NA	2,486 (1,325–4,103)
**Total deaths**	4,684 (1,213–12,514)	586 (86–2,498)	7,755 (2,626–19,116)

Annex 5: The probabilistic sensitivity analysis showing the influence of variables on the overall economic burden of typhoid fever in India

The direct costs in non-hospitalised cases, the overall mortality in non-hospitalised treatment-seeking cases, and the mortality and complications in hospitalised FQR cases are the most sensitive input parameters driving overall costs in the decision tree model.

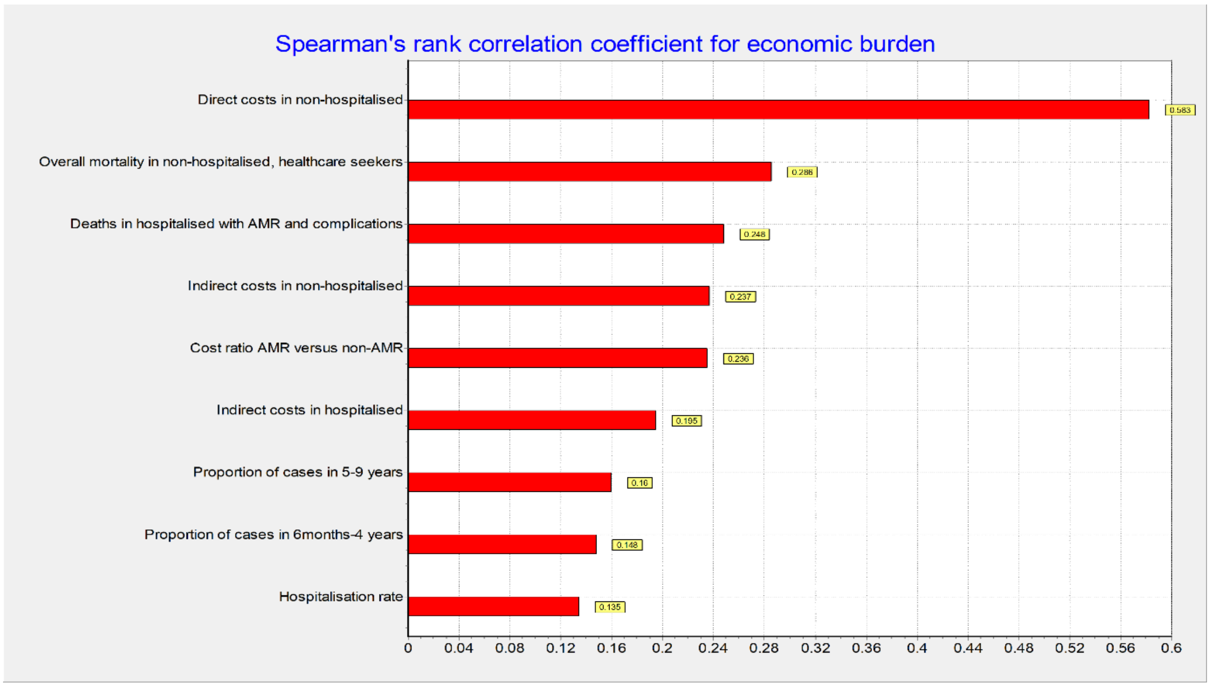

Annex 6: Age-specific economic burden of typhoid fever under two Primary scenarios

In the Primary Scenario, hospitalisations, complications, and deaths were redistributed by age using GBD 2021 patterns (2). In Primary Scenario B, we retained the age distribution of hospitalisations, complications, and deaths observed in SEFI surveillance data (2). The lighter shades in the figure shows 95% uncertainty intervals.

In the Primary Scenario, the highest economic burden was in the 5 to 9 year age group at INR 39.7 billion (95% UI: 23.1–70.0 billion; US$ 480 million, 95% UI: 280–847). The 6 months to 4 years group was followed at INR 36.5 billion (95% UI 22.3–62.9 billion; US$ 442 million, 95% UI 270–761). These accounted for 32% and 29% of the overall economic burden among treatment seekers, respectively (Figure 2). In Primary Scenario B, the burden was INR 46.3 billion (95% UI 8.0–79.0 billion; US$ 560 million, 95% UI 339–957) for 5 to 9 years, and INR 32.0 billion (95% UI 18.9–56.3 billion; US$ 387 million, 95% UI 228–682) for 6 months to 4 years. These made up 37% and 25% of the economic burden, respectively. Across both primary scenarios, children under 10 years consistently accounted for the largest share of economic burden.

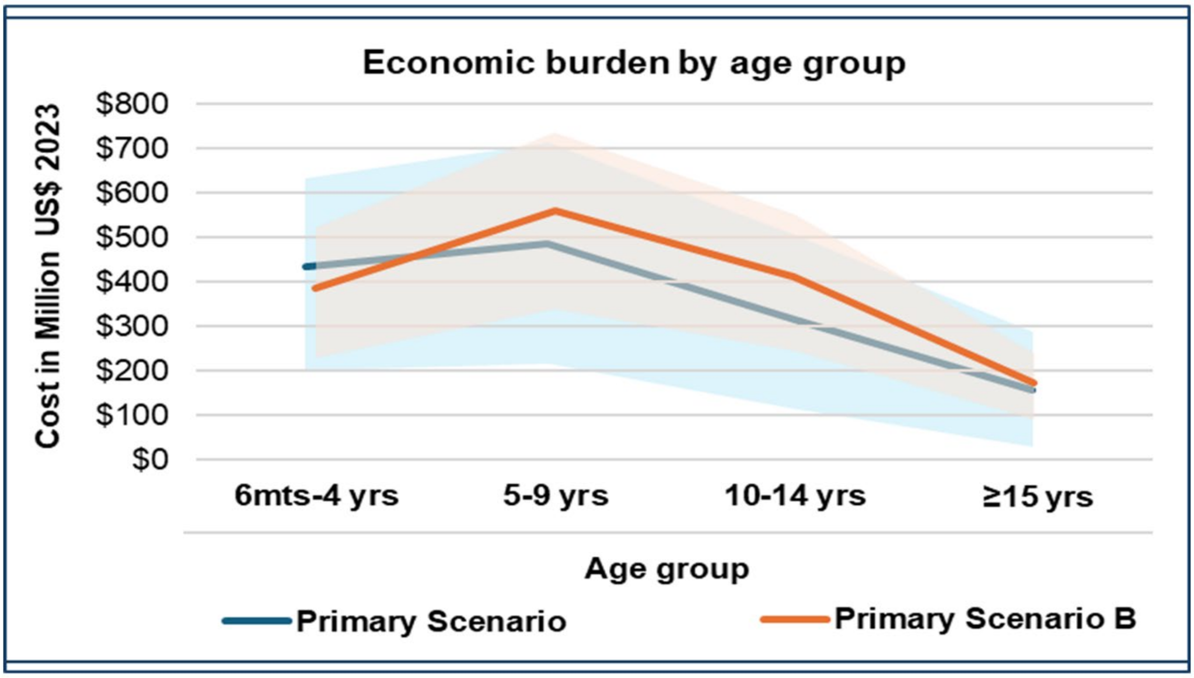

References

1. Kumar D, Sharma A, Rana SK, Prinja S, Ramanujam K, Karthikeyan AS, et al. Cost of Illness Due to Severe Enteric Fever in India. J Infect Dis. 2021;224(Supple 5):S540–S7.
2. Mogasale VV, John J, Sahai N, Ray A, Farooqui HH, Mogasale V, et al. Burden of typhoid fever and antimicrobial resistance in India (2023): a modelling study. Lancet Reg Health Southeast Asia. 2026;44:100714.
3. Office of the Registrar General and Census Commissioner, India. Census of India 2011: census tables. New Delhi: Ministry of Home Affairs, Government of India; 2011. Accessed April 19, 2023. Available from: https://censusindia.gov.in/census.website/ [Internet].
4. John J, Bavdekar A, Rongsen-Chandola T, Dutta S, Gupta M, Kanungo S, et al. Burden of Typhoid and Paratyphoid Fever in India. N Engl J Med. 2023;388(16):1491–500.
5. Cao Y, Karthikeyan AS, Ramanujam K, Raju R, Krishna S, Kumar D, et al. Geographic Pattern of Typhoid Fever in India: A Model-Based Estimate of Cohort and Surveillance Data. J Infect Dis. 2021;224(224 Supple 5):S475–S83.
6. Raju R, Kezia Angelin J, Karthikeyan AS, Kumar D, Kumar R R, Sahai N, et al. Healthcare Utilization Survey in the Hybrid Model of the Surveillance for Enteric Fever in India (SEFI) Study: Processes, Monitoring, Results, and Challenges. J Infect Dis. 2021;224(Supple 5):S529–S39.
7. Institute for Health Metrics and Evaluation (IHME). GBD Compare data visualization tool. Seattle: IHME, University of Washington; accessed September 23, 2024. Available from: https://vizhub.healthdata.org/gbd-compare/ [Internet]. 2024.
8. Mogasale VV, Zhang P, John J, Farooqui H, Ray A, Mogasale V, et al. Burden of antimicrobial resistance in culture-confirmed Salmonella Typhi isolates in India, 1977–2024: a systematic review and meta-analysis. SSRN preprint. 2025. Accessed June 25, 2025. Available from: https://ssrn.com/abstract=5317737 2025.
9. Bhutta ZA, Ahmed I, Als D, Radhakrishnan A, Qamar F, Stanaway J, et al. Antimicrobial resistance in typhoid: implications for policy and immunisation strategies. Presentation to the Strategic Advisory Group of Experts on Immunisation (SAGE); October 2017; Geneva, Switzerland. World Health Organisation. Accessed June 26, 2025. Available from: https://terrance.who.int/mediacentre/data/sage/SAGE_Docs_Ppt_Oct2017/4_session_typhoid/Oct2019_session4_Presentation3.pdf.
10. Samuel P, Njarekkattuvalappil SK, Kumar D, Raju R, Andrews JR, Kang G, et al. Case-Fatality Ratio of Enteric Fever: Estimates From Multitiered Surveillance in India. J Infect Dis. 2021;224(Supple 5):S517–S21.
